# The Case for Musical Instrument Training in Cerebral Palsy for Neurorehabilitation

**DOI:** 10.1155/2016/1072301

**Published:** 2016-10-27

**Authors:** Ana Alves-Pinto, Varvara Turova, Tobias Blumenstein, Renée Lampe

**Affiliations:** ^1^Research Unit for Paediatric Neuroorthopaedics and Cerebral Palsy, Orthopedic Department, Klinikum Rechts der Isar, Technical University of Munich, Ismaninger Str. 22, 81675 Munich, Germany; ^2^Markus Würth Stiftungsprofessur, Technical University of Munich, Munich, Germany

## Abstract

Recent imaging studies in cerebral palsy (CP) have described several brain structural changes, functional alterations, and neuroplastic processes that take place after brain injury during early development. These changes affect motor pathways as well as sensorimotor networks. Several of these changes correlate with behavioral measures of motor and sensory disability. It is now widely acknowledged that management of sensory deficits is relevant for rehabilitation in CP. Playing a musical instrument demands the coordination of hand movements with integrated auditory, visual, and tactile feedback, in a process that recruits multiple brain regions. These multiple demands during instrument playing, together with the entertaining character of music, have led to the development and investigation of music-supported therapies, especially for rehabilitation with motor disorders resulting from brain damage. We review scientific evidence that supports the use of musical instrument playing for rehabilitation in CP. We propose that active musical instrument playing may be an efficient means for triggering neuroplastic processes necessary for the development of sensorimotor skills in patients with early brain damage. We encourage experimental research on neuroplasticity and on its impact on the physical and personal development of individuals with CP.

## 1. Introduction

The central nervous system (CNS) has the ability to reorganize throughout life. This allows the CNS to adapt to changing environmental demands and to recover from injury [1]. This ability to adapt has spurred the search for new therapeutic approaches, especially in medical conditions resulting from damage to the CNS [2–4]. Also, advances in medical imaging, coupled with advances in basic neuroscience research, have stimulated the exploration of brain plasticity correlates of therapy/rehabilitation efficacy (e.g., [5, 6]). Whilst the potential for brain plasticity is widely acknowledged, the way in which structural and functional changes in the brain are optimally triggered towards rehabilitation of motor, cognitive, and/or executive functions, as well as the mechanisms through which neuroplasticity operates, remains unclear.

One of the medical conditions that may profit from this research is cerebral palsy (CP). In the foreground of this condition are disorders of motor function and posture resulting from injury to the CNS during development [7]. Patients may, however, also present sensory, cognitive, and attention deficits, as well as epilepsy. Despite this multisymptomatology, therapeutic programs in CP have concentrated mostly on orthopedic surgery and physical therapy to alleviate motor impairment and dysfunction [8]. Whilst this aims at giving the patients the tools to cope with demands in daily living, taking the other impairments into account in rehabilitation programs may increase the efficacy of rehabilitation training [9].

In recent years, increasing attention has been directed to the potential of music as driving vehicle for neuronal plasticity in general [10] but also in the context of neurological rehabilitation. This can be seen, for example, in the recent organization of the special research topic of “music, brain, and rehabilitation” [11]. Music has long played an important role in the therapy of children and teenagers with CP. The inclusion of music as therapy has been in many cases intended to increase patient's motivation or relaxation or, for example, as auditory feedback of movement [12] (passive/receptive use of music). The rhythmic properties of music have been also explored to tackle impaired gait patterns (e.g., [13, 14]) through rhythmic auditory stimulation. Despite the potential of music in CP, neuroscience-based evidence of the effect of music on the rehabilitation of these patients remains scarce. Furthermore, exploration of musical instrument training for the rehabilitation of upper limb motor function is almost nonexistent and in most cases assessed only from the point of view of psychological benefits (e.g., self-confidence) and the integration benefits it brings. The current review aims at collecting and summarizing scientific evidence that supports the potential benefit that music instrument training can have not only in the improvement of motor function in* patients with CP* but also with regard to their personal development.

We start by summarizing the main clinical characteristics of CP and alterations at the neuronal level that have been associated with specific deficits in CP. Next, we highlight the contribution of music and instrumental music to clinical rehabilitation in general with particular focus on motor disorders, selecting those that have implications for rehabilitation in CP. Finally, we review work investigating the effects of music and musical instrument training in CP. Recent reviews have covered the research on the effects of music in rehabilitation settings and on the hypothetical mechanisms underlying those effects. Our description is focused on the implications for rehabilitation in CP.

## 2. Characterization of Cerebral Palsy

A consensus definition of CP is as follows: “Cerebral Palsy describes a group of permanent disorders of the development of movement and posture, causing activity limitation, that are attributed to non-progressive disturbances that occurred in the developing fetal or infant brain. The motor disorders of CP are often accompanied by disturbances of sensation, perception, cognition, communication, and behavior, by epilepsy, and by secondary musculoskeletal problems.” [15]. We now address a few of the aspects of this definition that are relevant in view of the musical instrument-based training proposed later on in this paper. More information about these and other aspects of CP can be found in Rosenbaum and Rosenbloom [9].

The first aspect refers to limitations in gross motor function. These are critical for a diagnosis of CP. Rather than falling into enclosed descriptions, these limitations can vary, depending on the onset of injury, on the areas and extension of brain structures affected, and on the factors contributing to injury [7, 15]. Symptomatology is therefore heterogeneous among patients with CP. Nevertheless, in all cases, motor disabilities impact negatively on activities of daily living.

The second relevant aspect refers to the additional disturbances that often accompany motor limitations: disorders of sensation, perception, cognition, communication, behavior, and/or epilepsy [16–20]. These additional disturbances can occur primarily as a result of damage to brain structure and function [21–32] but can also occur in association with developmental limitations in motor function [9, 33]. Although occurrence of these additional disturbances is not decisive for the diagnosis of CP, they are often present and they increase the complexity of the patient's clinical picture and consequently of the choice of rehabilitation activities most effective for the patient. Importantly, however, the general consensus is that these additional disabilities need also to be addressed in the clinical management of CP, along with the motor deficits. Moreover, some deficits, for example, cognitive ones, further limit the choice of methods able to deliver effective rehabilitation.

Third, motor and additional disorders in CP result from damage to brain structures during development, pre-, peri-, or postnatally, but typically before the acquisition of skills (motor, sensory, perceptual, and cognitive skills). Consequently, rehabilitation in CP needs to promote the development of nonacquired skills. This is different from the case of disorders of movement that result from brain injuries occurring in adulthood or after motor development is completed, such as stroke or Parkinson's. In these cases rehabilitation is designed to promote the* recovery* of previously acquired skills. The skill-learning experience of patients with CP is, in comparison, very different and rehabilitation needs to focus on the* learning of “new” skills* [9] and needs to take into account the effect of age on the development of those motor, sensory, and cognitive skills [34].

Fourth, the “nonprogressive” character of the disturbances included in the definition refers to the static character of the encephalopathy, in that neuronal damage to the CNS is permanent and does not progress with time [9]. This “nonprogressive” character however does not extend to developmental changes during childhood and the neuronal, metabolic, and body changes during adulthood that patients with CP experience and that cannot be predicted. Hence, rehabilitation programs building on the plastic potential of the brain and the continuing adaption throughout life are likely to bring effective and long-term benefit to patients.

Clinical symptoms in CP are accompanied by abnormal neuroanatomical features, detected through neuroimaging in 80–90% of the cases [35] (Table 1). Most commonly reported cases are damage to white matter pathways, especially in cases of bilateral spastic CP and athetosis [35], and to both grey and white matter structures, more frequently reported in unilateral CP [36]. Other structural abnormalities mentioned include congenital malformations, atrophy, or enlarged ventricles and cerebrospinal fluid space abnormalities, among others. Corticospinal tract projections to peripheral muscles can also be affected in CP [37]. Commonly reported in functional magnetic resonance imaging and transcranial magnetic stimulation studies in unilateral CP patients is a reorganization of central neuronal pathways, with corticomotor projections from the ipsilateral (unaffected cortex) or both motor cortices to the lesioned hand [21, 22, 29, 30, 38–40] and contralateral projections to the unaffected hand [30]. CP patients present also a reduction in the volume of central motor structures, in comparison to controls [36, 39], reduced cortical activation during motor imagery tasks [32], and altered corticospinal pathway integrity and reduced white matter connectivity [24, 41, 42]. In addition to structural changes, movement-related neurophysiological activity is also altered in CP: magnetic resonance imaging studies have reported higher beta event-related desynchronization before movement initiation and lower gamma synchronization at the onset of movement [26], as well as lower alpha desynchronization during motor imaging [43] in patients with CP in comparison to controls.

However, structural alterations are not restricted to central motor tracts [21, 38, 40, 44, 47] but have also been shown to affect also sensorimotor pathways [23, 30, 31, 41, 42], with reorganization of the latter being dissociated from the reorganization in corticospinal tracts [22, 30]. Structural changes in sensorimotor pathways in CP are accompanied by altered somatosensory processing of tactile stimulation, with patients showing less extended cortical activation during tactile stimulation and lower magnitude of activation during tactile discrimination tasks [31]. Children with CP furthermore show, in comparison to typically developing peers, increased beta-activation [27] and altered activity in secondary sensory cortex in response to tactile stimulation [28]. The latter, together with the altered activation during discrimination tasks, which test the ability to distinguish between different stimuli, raises the possibility of abnormal central processing of sensory information in this clinical group and the possibility of impaired performance of motor tasks requiring the use of sensory information being due to altered sensory processing and sensorimotor interaction. Consistent with this hypothesis are the observed associations between behavioral measures of motor impairment and the degree of reorganization of sensorimotor pathways [22, 24, 26, 41]. All in all, it is now widely accepted that clinical symptoms in CP are often not exclusively due to disturbances of the motor pyramidal pathways but can often be due to deficits in connectivity within (and outside) sensorimotor networks and in integration and processing of sensorimotor information.

## 3. The Potential of Musical Instrument Training in the Development of Sensorimotor Interactions

Against this background, training methods that demand the use of sensory information in the context of a motor task may be beneficial in conditions where sensorimotor interactions are known to be impaired. With this aim in mind, musical instrument-supported training comes up as a prime candidate for training sensorimotor interactions in CP, since playing a musical instrument requires coordinating hand/finger movements with sensory—auditory, visual, and somatosensory—information (e.g., [10]); it involves continuous forward and backward transmission of information between different brain areas and between central and peripheral motor structures.

Effects of musical instrument training on brain structure and function have been especially investigated through imaging studies with trained musicians and nonmusicians. Studies have shown effects of long-term instrument musical training on brain plasticity both at the structural and at the functional level over several brain areas (for an in-depth review see [10, 48]). Long-term musical practice has been associated with anatomical differences in motor and auditory cortices (larger volume, greater thickness in musicians (e.g., [49, 50]), increased integrity of white matter (motor) pyramidal tracts [51], increased size of the corpus callosum [52], greater volume of cerebellum [53], and changes in multimodal integration areas (e.g., [54])). Interestingly, the type of instrument used in the training can influence the plastic mechanisms that can be measured (e.g., [54]). In addition to this, structural effects of musical training have been reported in children after 15 months of practice [55], and modulation of cortical motor outputs to the muscles involved in the performance of fine finger movements can be shaped after weeks of piano training in healthy adults [56]. Hence, musical instrument practice, and in particular piano training, has the potential to induce and promote structural and functional changes at the level of the CNS, and changes can take place at different ages (e.g., both children and adults are responsive) and at different timescales.

Beyond structural brain changes, also multimodal interactions seem to be promoted through musical instrument training and at very short time scales. In particular auditory-motor interactions have been observed in nontrained healthy adults shortly after learning a new audio-motor sequence [57] and after 20 minutes of piano training [58]. Multimodal integration seems also to be strongly promoted through musical instrument training [59], more than with auditory training only: music-associated measures of cortical plasticity were observed to be larger after musical instrument training in relation to training with auditory stimuli only [60, 61]. Given the bidirectional interaction between different sensory modalities, in particular between auditory and somatosensory systems [62], it is reasonable to assume that musical training, and musical instrument training in particular, will also have an impact on this interaction and on the neuronal processing of other sensory modalities [63].

This interaction between auditory and motor systems constitutes one of the possible means through which music-supported therapy (MST) can promote neurorehabilitation [64], especially in cases of motor disorders due to neurological damage [65] and in cases where both motor and sensorimotor networks are affected, as in CP. Even though the precise mechanisms through which damaged neuronal processes can be restored or improved remain unclear, numerous studies in the last years have shown that MST can positively influence the recovery, at least partially, of disturbed skills [59]. In one of these studies, stroke patients received regular training sessions playing MIDI piano and electronic drums (exercises adapted to individual capabilities) for three weeks [66]. After the training it was observed that both precision and smoothness of hand movements as well as the timing of movements had improved. It could also be observed that changes in oscillatory brain activity associated with motor planning (i.e., event-related desynchronization) and neuronal coherence were larger after MST than after conventional therapy [5]. MST was furthermore associated with changes in excitability in the motor cortex, derived with transcranial magnetic stimulation [6, 67], and with changes in the pattern of neuronal activation, measured with functional imaging, in a chronic stroke patient [6]. Once again, the fact that changes could be elicited in a patient 20 months after the injury [6] suggests that plasticity is not limited to the period immediately after damage but can still be triggered, albeit with less efficacy, months after injury.

As mentioned above the way in which MST triggers changes in neuronal processes that are damaged remains unclear, in particular which aspect of music—either the rhythmic component or the pitch structure component—is essential for triggering the recovery process. In fact, some rehabilitation programs based on music therapy use the rhythmicity or periodicity of music to influence, change, or* entrain* movements (for a review see [64]). In a study with hemiparetic stroke patients, rhythmic auditory stimulation was employed to train new gait patterns during the first three months after injury, by the training of walking following a rhythmic pattern, using a metronome or music tapes. Increases in gait velocity and in stride length and a reduction in leg-muscle EMG-amplitude were reported in these patients, in comparison to controls [68]. Similar training procedures have also been employed to train gait patterns in Parkinson's patients (e.g., [69]) and in children and adults with CP [13, 14, 70]. The fact that the training of walking using rhythmic sound properties with metronomes can influence walking kinematics in CP suggests that patients with CP can be responsive to MST-based training of movement.

## 4. Music-Supported Therapy in Cerebral Palsy

Music-based therapies are not new in the clinical management of CP. They have been typically employed to train more symmetrical and balanced gait patterns (see above) and as a vehicle for promoting motivation and emotional experience. The positive influence of music on the rehabilitation of patients with CP is reflected in the widespread use of music in group activities in rehabilitation and occupational day centers, where individuals typically play percussion instruments within group activities. Music is also often employed during relaxation exercises or in conjunction with other therapies with effects reported at the level of heart rate variability [71].

Less common in the rehabilitation of motor skills in CP however are MST-based methods and in particular the musical instrument training of hand motor function. In one of the few published studies, five adults with CP received a total of twelve sessions of musical instrument-based therapy on MIDI keyboard, twice a week for six to nine weeks [72]. The playing speed on the keyboard increased with the training to values closer to those obtained from a control group of able-bodied individuals. In another study eighteen young people (6–16 years) with CP received individual piano training with a professional piano teacher twice a week for 18 months [73]. In this case a reduction in the variability of keystroke timing was interpreted as indicating an improvement in the uniformity of keystrokes with the piano training. In an associated study, potential neuronal correlates of the effects of piano training were investigated by analyzing effective connectivity between the cerebellum and primary motor cortex in a group of ten young people with CP and comparing these results with those from a similar group of six that had received conventional therapy only [73]. An increase in effective connectivity from the left primary motor area to the right cerebellum in the group that received the training relative to the control group was interpreted as indicating a neuronal plastic effect resulting from piano training.

The effects of musical instrument training may however likely extend beyond motor areas and function. By recruiting the entire CNS it may likely, more than any other simple motor training, influence more strongly sensorimotor interactions leading to coordinated motor responses.

## 5. Final Remarks

To our knowledge the study of Alves-Pinto et al. [73] is the only study that has attempted to investigate the neuronal correlates of musical instrument training in CP and in particular in association with the training of hand motor function. Such studies are important to confirm that functional rehabilitation is not due to chance but that the therapy/training has introduced changes in the internal mechanisms underlying motor function [74]. However the validity of results can deliver false inferences if the correct experimental controls are not observed [74, 75]. One of the difficulties for studies investigating neuroplasticity in CP (e.g., functional imaging) lies in the heterogeneity of this clinical group, with patients differing greatly in the symptoms presented, in the underlying brain damage, and in the onset of injury. Ways to overcome these difficulties are becoming available, especially for imaging and neurophysiological data collected in patients with CP [74].

Besides the clinical heterogeneity in CP, additional challenges lie in the disabilities concomitant to motor impairments that are often present, namely, the learning difficulties. Besides influencing the range of experimental tests that can detect functional change induced by training, they are likely to have implications in the way musical instrument training can effectively support patients' rehabilitation. Individualized training that takes into account the specific impairments and development stage of each patient, as well as the individual training period required for plastic changes to occur, may in this case be advised in order to maximize benefit. Capturing these changes will require appropriate experimental methodology. As mentioned above, music already plays a central role in the management of patients with CP, via music therapy-based group activities that promote personal and emotional enrichment, socializing, and relaxation. For the reasons presented above, musical instrument training, particularly* active *piano training on an* individual* basis, has the potential to trigger and promote the neuronal plastic changes and sensorimotor interactions required for rehabilitation in CP. However, experimental research is needed to validate, support, and guide the optimal use of musical instrument training in the learning of sensorimotor abilities in patients with impairments due to early brain damage.

## Figures and Tables

**Table 1:** Main results of imaging studies regarding alterations in neuronal structure and neuronal activity in CP. CP: cerebral palsy, TD: typically developing, TMS: Transcranial Magentic Resonance Imaging, EMG: Electromyography, fMRI: functional Magentic Resonance Imaging, SEP: Somatosensory Evoked Potential, MEG: magnetic resonance imaging, DTI: Diffusion Tensor Imaging, and ERP: Event-Related Potential.

Study (1st author, year)	CP group	Control group	Experimental methods	Main results
Farmer (1991) [38]	4 unilateral CP, 2–5 yrs	10 age-matched TD children	TMS, EMG.	Projections from ipsilateral cortex to impaired hand in CP.

Cooper (1995) [16]	9 hemiplegic, 4.4–18 yrs	41 age-matched TD teenagers	SEP, hand motor function tests, clinical sensory tests (stereognosis and proprioception).	Bilateral sensory deficits in hemiplegic CP. SEP correlated with motor function.

Thickbroom (2001) [30]	7 unilateral CP, 17–57 yrs		TMS, fMRI.	Unaffected hand: contralateral projection. Affected hand: either ipsilateral or bilateral projection. Contralateral projection with passive movement.

Staudt (2004) [44]	34 unilateral CP, 5–27 yrs		TMS and fMRI during simple hand movements.	In 16 patients the paretic hand was controlled via ipsilateral corticospinal projections. Motor dysfunction correlated with timing of brain lesion.

Guzzetta (2007) [22]	12 unilateral, 10–28 yrs		SEP, structural MRI, functional MRI during sensory stimulation and during hand movement, TMS, motor function test.	Perilesional reorganization of somatosensory function in 11 patients. Reorganization of motor function in the ipsilateral hemisphere in 5 individuals → dissociation motor-somatosensory. Correlation between fMRI activation and sensory deficit.

Bleyenheuft (2007) [40]	12 unilateral CP, 10–16 yrs	12 age-matched teenagers	Structural and diffusion MRI.	Correlation between corticospinal dysgenesis and upper limb impairment. DTI results correlated with stereognosis and digital and manual dexterities and abilities.

Hoon (2009) [24]	21 spastic diplegia, 4 with spastic quadriplegia, 2 with hemiplegia, 1 with ataxic/hypotonic CP, 16 months–13 yrs	35 age-matched, TD children	DTI, perceptual tests of touch, proprioception, tests of strength and spasticity.	All children with CP had periventricular white-matter injury. Injury in posterior thalamic radiation and descending corticospinal tracts. Injury on the posterior thalamic pathways correlated with touch threshold, proprioception, motor severity.

Wingert (2010) [31]	10 spastic diplegia, 10–34 yrs	10 age-matched participants	fMRI during tactile perceptual and discrimination tasks.	Relative to controls the CP group showed less extensive cortical activation and lower magnitude of activation in areas associated to somatosensation and motor and attention-relevant cortical areas.

Nevalainen et al. (2012) [45]	8 hemiplegic, mean age: 14.5 yrs	8 age-matched TD children	MEG, tests of somatosensory perception and discrimination.	Ipsilateral (i.e., ipsilesional) somatosensory representation but altered SEP bilaterally. Altered morphology of median nerve evoked fields.

Rose (2011) [39]	16 unilateral CP		Diffusion and structural MRI.	Reduced volume of ipsilesional precentral gyrus. Decreased ipsilesional corticospinal and corticothalamic pathways in comparison with contralesional ones. Sensorimotor thalamic projections better correlated with paretic hand motor function than corticospinal projections.

Maitre et al. (2012) [28]	8 unilateral CP, 5–10 yrs		ERP to sham or air puff stimulation. Quality of upper extremity skill test. Two-point discrimination test. Measurement of stereognosis and grip strength.	No differences in the responses associated with primary sensory cortex between the affected and unaffected hands. Differences in responses from secondary sensory cortex (larger N140 on the unaffected hemisphere). Larger latency of ipsilateral compared to contralateral P300.

Kurz (2014) [26]	11 spastic diplegic or hemiplegic CP, 10–18 yrs	11 age-matched TD children	Oscillatory activity (MEG) evoked by tactile stimulation of the foot. Measurement of errors during a match-to-target task with the foot.	Desynchronization in somatosensory cortices in CP relative to synchronized activity in controls. Degree of synchronization negatively correlated with number of errors in match-to-target task.

Holmefur et al. (2013) [46]	32 unilateral CP, 18 months–8 yrs		Computerized tomography, structural MRI.	Hand function predicted by pattern of damage and white matter damage. Better performance associated with absence of lesions in the thalamus or basal ganglia.

Englander (2013) [42]	17 spastic bilateral CP, 1–5 yrs		Diffusion MRI.	Differences in total white matter connectivity throughout the brain and long-range connectivity between severe and moderate CP, but no differences in short-range connectivity. Reduction in white matter connectivity included sensorimotor and nonsensorimotor regions.

Daly et al. (2015) [43]	Upper, lower, or both limbs affected, 20–58 yrs	12 age-matched able-bodied adults	EEG: alpha ERD, phase synchrony, phase dynamics during motor imagery.	Less ERD and less phase locking over motor cortex in CP.

Pannek (2014) [41]	50 unilateral CP, 5–16 yrs	17 age-matched TD children and teenagers	Diffusion MRI, assisting hand assessment.	Pathway integrity and connectivity reduced in CP compared to controls. Positive relationship between performance in bimanual tasks and integrity and connectivity in corticospinal and thalamocortical pathways.

Scheck (2014) [36]	72 unilateral CP, 5–17 yrs	19 age-matched TD children and teenagers	Structural MRI.	Reduction in grey matter volume in several subcortical structures in CP compared to controls.

Chinier (2014) [32]	20 unilateral CP		Functional MRI of motor imagery task.	Reduced activation for patients with right brain damage, with bilateral distribution of activation.

Kurz (2014) [26]	9 spastic diplegia, 4 hemiplegia, mean age: 14 yrs 3 months	13 age-matched TD children	MEG during extension of knee joint.	Higher beta event-related desynchronization before the onset of movement and lower gamma event-related synchronization at the onset of movement in CP relative to controls.

Kurz (2015) [27]	4 spastic diplegia, 2 hemiplegia, 2 quadriplegia, 2 spastic, 12–18 yrs	8 aged matched TD teenagers	Oscillatory activity (MEG) in response to tactile stimulation of the hand.	Theta-alpha oscillations intact but increased beta activity in children with CP compared to controls, suggesting altered somatosensory processing.
